# Decoding Coherent Patterns from Arrayed Waveguides for Free-Space Optical Angle-of-Arrival Estimation

**DOI:** 10.3390/s25237231

**Published:** 2025-11-27

**Authors:** Jinwen Zhang, Haitao Zhang, Zhuoyi Yang

**Affiliations:** State Key Laboratory of Precision Space-Time Information Sensing Technology, Department of Precision Instrument, Tsinghua University, Beijing 100084, China; jinwen-z21@mails.tsinghua.edu.cn (J.Z.); zy-yang22@mails.tsinghua.edu.cn (Z.Y.)

**Keywords:** angle-of-arrival estimation, arrayed waveguide, coherent mode decoding, convolutional neural network, attention mechanism

## Abstract

This paper presents a novel free-space optical Angle-of-Arrival (AOA) estimation method based on arrayed waveguide coherent mode decoding, aiming to surpass the inherent limitations of traditional AOA detection technologies, which face significant challenges in achieving miniaturization, low complexity, and high reliability. The method utilizes the AOA-related phase differences generated by the propagation and interference of incident light in an arrayed input waveguide, forming multi-beam interference fringes at the output end of the slab waveguide. This pattern is then sampled by an arrayed output waveguide to produce an intensity sequence, which is then fed into a trained CNN–Attention Regressor for AOA estimation. This study innovatively applies the method to decoding the spatial angular information of optical signals. Simulation results demonstrate the exceptional performance of our approach, achieving a Mean Absolute Error (MAE) of 0.0142° and a Root Mean Square Error (RMSE) of 0.0193° over a 40° field of view. This precision is significantly superior to conventional peak–linear calibration methods and other common neural network architectures, and exhibits remarkable robustness against simulated phase noise and manufacturing tolerances. This research demonstrates the powerful synergy between integrated photonics and deep learning, paving the way for a new class of highly integrated, robust, and high-performance on-chip optical sensors.

## 1. Introduction

Optical Angle-of-Arrival (AOA) estimation is a critical technique for spatial parameter sensing playing a pivotal role in fields such as Free-Space Optical (FSO) communication [1,2,3], LiDAR (Light Detection and Ranging) [4,5,6], and Laser Warning Systems (LWS) [7,8]. Its performance directly impacts the system’s capabilities in areas such as beam alignment, target localization, and threat identification [2,3,6,7]. With the increasing demand for device miniaturization, wide field of view, high precision, and high integration in modern applications [2,3,6,7], the limitations of traditional angle detection technologies have become increasingly prominent.

The landscape of optical AOA detection encompasses several distinct approaches. One category of techniques relies on discrete optical components. For example, lens-based systems determine the AOA by calculating the centroid of a light spot on a photosensitive detector, such as a Four-Quadrant Detector (QPD) [3,9]. Similarly, methods based on detector arrays have been widely explored, inferring the AOA by measuring the differential incident optical flux across array elements positioned at different spatial locations [3,10]. While effective, these approaches generally depend on bulky and structurally complex macroscopic systems, posing significant challenges for miniaturization and seamless integration with modern photonic circuits.

To address these integration challenges, more advanced array-centric technologies have been investigated. Optical Phased Arrays (OPAs) can be utilized for optical AOA detection, where the receiver employs programmable angular selectivity to collect light from a specific angle while suppressing off-axis signals [11,12]. However, OPAs typically require active electrical control systems for beam steering, meaning they are not fully passive devices, and their on-chip integration remains complex. Many advanced concepts from the radio-frequency (RF) domain, while well-established, have not been transferred to the optical domain. For instance, a space-time modulated metasurface has been proposed for AOA estimation by measuring the amplitude imbalance between harmonic frequencies of the reflected field [13]. Similarly, leveraging time delay is a well-established principle in RF systems. This has been adapted for AOA detection, where multiple spatially distributed receivers measure the relative time delay between different signal channels to derive the angle [14,15]. On the integrated photonics front, a method utilizing a PIC-based star coupler has been demonstrated to achieve an optical Fourier transform, replacing a conventional lens for two-dimensional (2D) AOA detection [16,17,18]. While this approach offers a significant reduction in system Size, Weight, and Power (SWaP) [17], these advanced techniques collectively highlight a persistent challenge: they are primarily validated in the RF domain with non-trivial transitions to optical frequencies, or have not yet been tailored for passive, high-precision AOA decoding.

A review of the existing landscape reveals that current AOA detection methods are either constrained by the bulkiness of discrete components or rely on active, complex systems, with some advanced concepts yet to be demonstrated effectively and passively in the optical domain. This highlights a clear demand for a solution that is compact, passive in its optical detection stage, and amenable to chip-scale integration.

In parallel, the field of integrated photonics has revolutionized optical communication and sensing, celebrated for its advantages in miniaturization, stability, and potential for mass production [19,20]. Applying the principles of this powerful platform to the challenge of optical AOA detection, therefore, presents a compelling yet underexplored research avenue. Among the rich library of integrated photonic devices, the Arrayed Waveguide Grating (AWG) is a cornerstone component for Wavelength Division Multiplexing (WDM), widely used for multi-channel signal processing and high-precision spectral analysis due to its low loss, high channel capacity, and excellent channel isolation [21,22,23]. The working principle of an AWG is based on the phase accumulation from path length differences in its arrayed waveguides—light at different wavelengths accumulates different phases, enabling selective separation through interference [21]. Its high degree of tunability in geometric parameters and phase control endows the structure with significant design freedom [24]. Despite its widespread use, the potential of the arrayed waveguide for spatial information sensing, such as optical AOA detection, has remained unexplored. Given the phase-sensitive nature of the arrayed waveguides, it is theoretically plausible to convert the spatial phase front of an incident light field into a measurable intensity distribution, offering a novel pathway for highly integrated, mechanical-scan-free angle measurement. However, this direction has several research gaps, necessitating a systematic evaluation of the feasibility of arrayed waveguide devices for optical AOA detection—from theoretical modeling and device design to performance verification—to provide a methodological foundation for future implementation.

To bridge this identified research gap and harness the untapped potential of arrayed waveguides for spatial sensing, this paper proposes and numerically validates an optical AOA detection method based on arrayed waveguides. This work extends the function of an on-chip arrayed waveguide from a traditional wavelength selector/demodulator to a decoder of spatial angular information. The proposed method unfolds in four key stages. Firstly, incident free-space light is coupled into a high fill-factor arrayed waveguide, generating a phase and amplitude distribution along the propagation path that is dependent on AOA. Secondly, within the slab waveguide, these multiple beams undergo coherent superposition to form spatial interference fringes that are coupled with the incident angle. Then, an arrayed output waveguide at preset positions performs mode coupling and intensity sampling on the light field at the output plane of the slab waveguide, yielding a spatial intensity sequence. Finally, this intensity sequence is fed into a trained CNN–Attention regressor [25,26,27,28,29], which directly regresses the incident angle, ensuring high accuracy and robustness in the model’s predictions.

The main contributions of this paper can be summarized as follows:

- A novel application for the arrayed waveguides—optical AOA detection—is conceptually proposed and demonstrated. By fully exploiting its inherent phase sensitivity and integrated photonic platform, this approach enables miniaturized, high-precision angle sensing, extending its functionality beyond conventional wavelength division multiplexing.
- A complete hybrid detection architecture is designed that synergistically combines the arrayed waveguides with the powerful CNN–Attention regressor, creating an end-to-end AOA detection pipeline.
- Through comprehensive numerical simulations, the proposed method has been validated. The results show that for angle prediction over a 40° field of view, the method achieves a Mean Absolute Error (MAE) of 0.0142° and a Root Mean Square Error (RMSE) of 0.0193°. These findings demonstrate the method’s high accuracy and robustness in AOA estimation, providing a solid methodological basis for future experimental implementation.

## 2. Materials and Methods

This section details the design of the arrayed waveguide structure, the optical field simulation method, the noise model, and a convolutional neural network (CNN) combined with a self-attention mechanism (CNN–Attention Regressor) architecture used for AOA estimation.

The arrayed waveguide structure investigated in this paper is designed to operate primarily in a single plane. The core physical principle—converting the AOA into a spatial shift of the interference peak—is predominantly a one-dimensional (1D) phenomenon along the axis of the Rowland circle. To efficiently validate this fundamental concept and generate the large dataset required for training the deep learning model, we have simplified the two-dimensional (2D) optical simulation problem into a 1D model. This simplification significantly reduces computational complexity, making the extensive numerical experiments feasible.

While this 1D model captures the essential beam-steering physics and allows for a robust proof-of-concept demonstration, we acknowledge that it represents a first-order approximation of a real-world 2D device. The potential impacts and limitations arising from this simplification, such as resolution asymmetry and polarization-dependent effects, are non-trivial. A detailed discussion of these factors and their implications for practical implementation is provided in the Discussion section (Section 4). Therefore, the subsequent simulation analysis based on this 1D model serves as a foundational study to establish the method’s viability, with its conclusions providing a strong baseline for future 2D modeling and experimental verification.

### 2.1. Waveguide Structure and Optical Propagation

**Figure 1:**
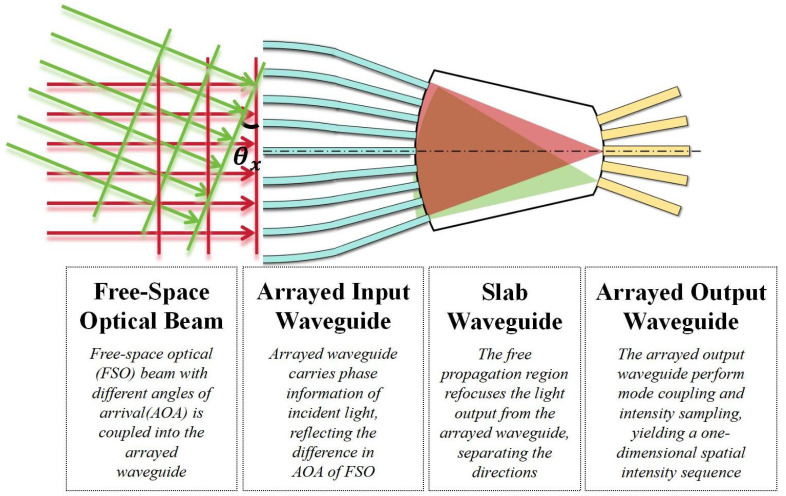
Basic structure of the arrayed waveguide model for Angle-of-Arrival (AOA) detection. The red lines represent normally incident beams, the green lines represent obliquely incident beams, the blue structure is the arrayed input waveguide, and the yellow structure is the arrayed output waveguide. Both are arranged equidistantly on opposite sides of the slab waveguide, following the Rowland circle geometry.

In this study, we construct an arrayed waveguide structure that converts the spatially distributed phase information corresponding to the AOA of free-space light into spatially distributed intensity information. As shown in Figure 1, the structure uses arrayed waveguides, with elements arranged equidistantly on an arc of radius R = 2r, to collect the phase information of the incoming free-space light. Multiple beams interfere within a slab waveguide, causing beams with the same AOA to focus at the output plane of the slab waveguide. An array of output waveguides, arranged equidistantly around a Rowland circle of radius r, samples the intensity of the output optical field, yielding a spatial optical intensity sequence that is correlated with the AOA.

The Rowland circle geometry describes the inherent focal curve of our device [21,30]. For different Angles of Arrival, the focal points trace this circle, not a straight line. Placing the output waveguides on this curve is therefore a fundamental requirement to minimize optical aberrations and ensure optimal focusing.

#### 2.1.1. Basic Assumptions

In this research, we build an arrayed waveguide AOA estimation architecture on a simulation platform for optical field simulation. The simulation environment is based on rigorous electromagnetic field theory to ensure the physical accuracy of the calculation results.

It is assumed that the input is a tilted Gaussian beam with a finite beam waist [31], incident from a certain position in space onto the center of the arrayed input waveguide structure. Its complex amplitude distribution can be expressed as

(1) $$E_{\mathrm{tilt}}^{2}\left(x,y,z\right)=\exp\left[-\frac{\left(\eta^{2}+\xi^{2}\right)}{\omega^{2}\left(\chi \right)}\right]\times \exp\left\{-i\times k\left[\frac{\left(\eta^{2}+\xi^{2}\right)}{2\Delta R}+\chi \right]-\Delta \psi \right\},$$

with

(2) $$\eta =\cos\theta_{x}x+\sin\theta_{y}\sin\theta_{x}y-\sin\theta_{x}\cos\theta_{y}z-x_{0},$$

(3) $$\xi =\cos\theta_{y}y+\sin\theta_{y}z-y_{0},$$

(4) $$\chi =\sin\theta_{x}x-\sin\theta_{y}\cos\theta_{x}y+\cos\theta_{x}\cos\theta_{y}z,$$

(5) $$\Delta R=Z_{0}\left(\chi /Z_{0}+Z_{0}/\chi \right),$$

(6) $$\Delta \psi =\arctan\left(\chi /Z_{0}\right),$$

(7) $$w\left(\chi \right)=w_{0}\sqrt{1+{\left(\chi /Z_{0}\right)}^{2}},$$

(8) $$z_{0}=\frac{\pi \omega_{0}^{2}}{\lambda },$$

Assuming the waveguide operates under the weakly guiding approximation, the Effective Index Method (EIM) is used to calculate the effective refractive index of the modes [32]. This method decomposes the three-dimensional waveguide structure into two-dimensional slab waveguide problems. The effective refractive index of the guided modes is obtained by solving the eigenvalue equation:

For TE modes, the eigenvalue equation is

(9) $$\kappa h=m\pi +\arctan\left(\frac{p_{0}}{\kappa }\right)+\arctan\left(\frac{p_{2}}{\kappa }\right),$$

For TM modes, the eigenvalue equation is

(10) $$\kappa h=m\pi +\arctan\left(\frac{n_{1}^{2}}{n_{0}^{2}}\frac{p_{0}}{\kappa }\right)+\arctan\left(\frac{n_{1}^{2}}{n_{2}^{2}}\frac{p_{2}}{\kappa }\right),$$

with

(11) $$\kappa ={\left(k_{0}^{2}n_{1}^{2}-\beta^{2}\right)}^{1/2},$$

(12) $$p_{0}={\left(\beta^{2}-k_{0}^{2}n_{0}^{2}\right)}^{1/2},$$

(13) $$p_{2}={\left(\beta^{2}-k_{0}^{2}n_{2}^{2}\right)}^{1/2},$$

where $n_{1}$, $n_{2}$, and $n_{0}$ are the refractive indices of the core, upper cladding, and substrate, respectively.

The simulation employs a dispersion model based on the Sellmeier equation to describe the refractive index of materials at specific wavelengths [33]. For the waveguide material, the following third-order Sellmeier equation is used.

(14) $$n^{2}\left(\lambda \right)=1+\frac{s_{1}\lambda^{2}}{\lambda^{2}-\lambda_{1}^{2}}+\frac{s_{2}\lambda^{2}}{\lambda^{2}-\lambda_{2}^{2}}+\frac{s_{3}\lambda^{2}}{\lambda^{2}-\lambda_{3}^{2}},$$

where the Sellmeier coefficients $s_{1}$, $s_{2}$, $s_{3}$ and the resonant wavelengths $\lambda_{1}$, $\lambda_{2}$, $\lambda_{3}$ are either constants or polynomial functions of temperature.

#### 2.1.2. Optical Field Propagation

**Figure 2:**
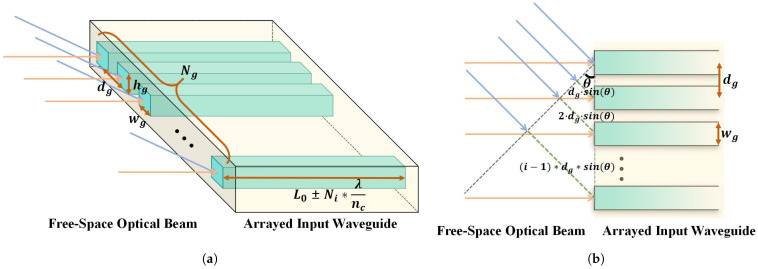
Structure and principle of the arrayed input waveguide. (**a**) Schematic of the arrayed input waveguide structure and its parameters. The light-yellow structure is the upper cladding and substrate, and the light-blue is the waveguide core. Here, $N_{i}$ is a positive integer and $L_{i}=L_{0}\pm N_{i}*\frac{\lambda }{n_{c}}$ indicates that the optical path difference caused by the length difference between different channels of the arrayed input waveguide should be an integer multiple of the wavelength. (**b**) Mechanism of phase difference generation for beams incident at different AOAs. The light-yellow structure is the upper cladding and substrate, and the light-blue is the waveguide core.

The arrayed input waveguide structure is shown in Figure 2a, where the light-yellow structure represents the upper cladding and substrate, and the light-blue structure is the waveguide core. The energy of the incident light beam is coupled into and received by the cores of the arrayed input waveguides. The meanings of the relevant structural parameters are given in Table 1. It is important to note that $N_{i}$ in the figure is any positive integer, and $L_{i}=L_{0}\pm N_{i}*\frac{\lambda }{n_{c}}$ represents the waveguide length of the *i*-th channel of the arrayed input waveguide.

For a plane wavefront incident at a certain oblique AOA, there exists an optical path difference ΔL$=d_{g}$sinθ between adjacent arrayed waveguides, as shown in Figure 2b. This path difference is determined by the geometric distance $d_{g}$ between the centers of two waveguides and the sine of the incident angle θ, resulting in a phase difference $\Delta \varphi =2\pi \frac{d_{g}}{\lambda }\sin\theta$. The waveguide length of the central channel of the arrayed input waveguide is $L_{0}$. For a given wavelength, to ensure that the arrayed input waveguide structure itself does not introduce any additional phase difference, the lengths of the arrayed input waveguides should be $L_{i}=L_{0}\pm N_{i}*\frac{\lambda }{n_{c}}$. In this way, the optical path difference between different channels of the arrayed input waveguide structure is related only to the incident angle and the spacing between waveguide centers, thus achieving the collection of phase information related to the AOA.

After the optical field is collected by the arrayed input waveguide, it continues to propagate through the slab waveguide. The propagation process of the optical field in the waveguide is primarily simulated using the Rayleigh-Sommerfeld diffraction integral [34]. This method, based on scalar diffraction theory, simulates the propagation from the near-field to the output plane. The integral formula is as follows:

(15) $$V\left(x,\;y,\;z\right)=\int \int V\left(x^{\prime },\;y^{\prime },\;0\right)\left[\frac{1}{2\pi }\frac{z}{R}\left(1-ikR\right)\frac{\exp(ikR)}{R^{2}}\right]dx^{\prime }dy^{\prime },$$

where V(x, *y*, z) and $V(x^{\prime }$, $y^{\prime }$, 0) represent the complex amplitude distributions at the output and input planes, respectively, $R=\sqrt{{\left(x-x^{\prime }\right)}^{2}+{\left(y-y^{\prime }\right)}^{2}+z^{2}}$ is the propagation distance, and k=2π/λ is the wavenumber.

This method provides good accuracy in both near-field and far-field regions, making it suitable for diffraction calculations in arrayed waveguide structures.

**Figure 3:**
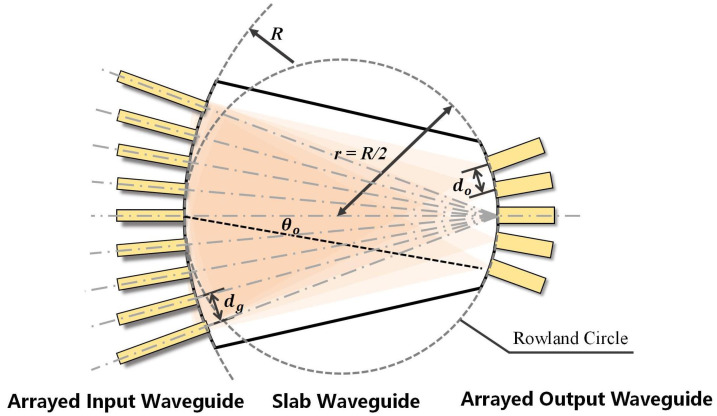
Structural details of the slab waveguide in the arrayed waveguide AOA detection model.

The arrayed waveguide structure is shown in Figure 3, which illustrates the internal layout of the model and the relationships between its components [21]. It can be seen that the arrayed input waveguide and the arrayed output waveguide are distributed on opposite sides of the slab waveguide, arranged equidistantly on two arcs of the slab waveguide. The central axes of all channels of the arrayed input waveguide point towards the center of the output waveguide structure, and the propagation length is the Rowland circle diameter R.

#### 2.1.3. Structural Parameter Design

For constructive interference to occur at a specific position on the output facet of the slab waveguide, the phase difference for beams from different channels of the arrayed input waveguide arriving at the same focal point must be an integer multiple of 2π. This satisfies the grating equation:

(16) $$\beta_{c}L_{0}+\beta_{s}\left(R-\frac{d_{g}}{2}\sin\theta_{o}\right)\;=\beta_{0}d_{g}\sin\theta +\beta_{c}L_{0}+\beta_{s}\left(R+\frac{d_{g}}{2}\sin\theta_{o}\right)-2m\pi ,$$

where $\beta_{0}$, $\beta_{s}$ and $\beta_{c}$ are the propagation constants of the beam in air, in the slab waveguide, and in the arrayed waveguides, respectively. The parameter θ represents the angle between the input beam and the central axis of the arrayed waveguide structure, i.e., the AOA. The parameter $\theta_{o}$ represents the angle between the output waveguide and the axis of the slab waveguide’s output facet. Other parameters and their meanings are given in Table 1.

We note that the terms $\beta_{c}L_{0}$ and $\beta_{s}R$ cancel on both sides. Rearranging the remaining terms and using the relations $\beta_{0}=\frac{2\pi }{\lambda }$ and $\beta_{s}=\frac{2\pi n_{s}}{\lambda }$, where $n_{s}$ is the effective refractive index of the slab waveguide, we obtain the basic grating equation for the arrayed waveguide AOA detection model:

(17) $$d_{g}sin\theta +n_{s}d_{g}sin\theta_{o}=m\lambda ,$$

- Angular and Positional Sensitivity to AOA

The grating equation shows that a change in the incident angle will lead to a difference in the beam’s output angle, ultimately causing it to focus at a different position. Therefore, an angular-AOA sensitivity equation can be formulated to describe the relationship between the angle of the diffraction maximum and the AOA. Similarly, a positional-AOA sensitivity equation can be used to describe the correspondence between the focal position of the output beam and the AOA.

By differentiating the grating equation with respect to the incident angle θ, we get

(18) $$d_{g}\cos\theta +n_{s}d_{g}\cos\theta_{o}\frac{d\theta_{o}}{d\theta }=0,$$

Thus, we can obtain the angular-AOA sensitivity equation:

(19) $$\frac{d\theta_{o}}{d\theta }=-\frac{\cos\theta }{n_{s}\cos\theta_{o}}$$

Similarly, the positional-AOA sensitivity equation can be derived:

(20) $$\frac{dx}{d\theta }=R\frac{d\theta_{o}}{d\theta }=-\frac{R\cos\theta }{n_{s}\cos\theta_{o}}$$

The angular resolution of the device is influenced by structural parameters such as the spacing and refractive index of the arrayed waveguides. For instance, a larger waveguide spacing and a smaller refractive index can effectively increase the separation between the maxima of interference patterns formed by beams with different incident angles, thereby enhancing angular resolution. Concurrently, a larger clear aperture can produce sharper and finer interference fringes, further improving the accuracy and robustness of angle estimation.

- Angular Detection Range

We define Δθ as the difference between the angles of two incident beams of different directions from adjacent diffraction orders that focus at the same final position. Starting from the grating equation, when the difference in the direction angle of the input beam is Δθ the m-th diffraction order of the central wavelength coincides with the (m + 1)-th order:

(21) $$d_{g}\sin\left(\theta +\Delta \theta \right)+n_{s}d_{g}\cos\theta_{o}=\left(m+1\right)\lambda ,$$

(22) $$d_{g}\sin\theta +n_{s}d_{g}\cos\theta_{0}=m\lambda ,$$

Subtracting the two equations gives

(23) $$\Delta \theta =sin^{-1}\left(\frac{\lambda_{c}}{d_{g}}+\sin\theta \right)-\theta ,$$

From the analysis of the above formula, it is known that the angular detection range is related to the incident angle, wavelength, and the spacing of the arrayed input waveguides. Furthermore, when $\theta =sin^{-1}\left(-\frac{\lambda_{c}}{2d_{g}}\right)$ the angular detection range has an extremum. At this point, it can be calculated that

(24) $$\Delta \theta =2sin^{-1}\left(\frac{\lambda_{c}}{2d_{g}}\right),$$

This formula can be used to approximate the angular detection range.

### 2.2. Angle Estimation Methods

For the fringe interference light field at the output of the planar waveguide, this study investigates the use of arrayed output waveguides for mode coupling. Ultimately, through intensity sampling, the light field coupling efficiency on different waveguide channels is calculated, and the light intensity received by different channels is outputted to obtain a light intensity sequence of length $N_{o}$. In response to the intensity sequence signal generated by simulation, this study proposes an end-to-end regression method based on a CNN–Attention model for angle estimation and compares its performance with the conventional peak–linear calibration method as well as other deep learning architectures.

#### 2.2.1. CNN–Attention Regressor Method

**Figure 4:**
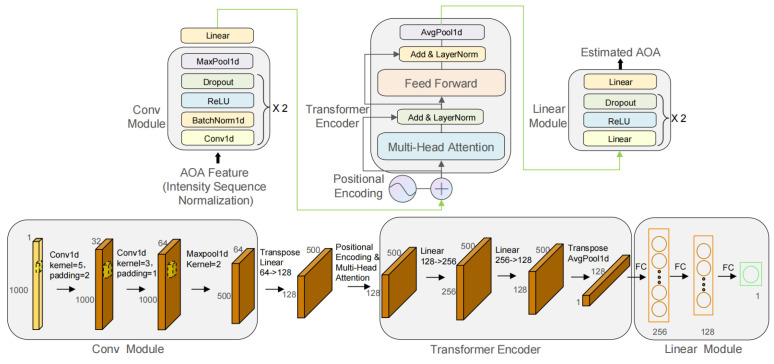
The architecture and data flow diagram of the proposed CNN–Attention regressor.

This paper adopts a hybrid neural network architecture for angle regression, designed to accurately predict the AOA of incident free-space light from fringe data. This network combines the feature extraction capabilities of a convolutional neural network with the sequence modeling advantages of a multi-head self-attention mechanism. The overall architecture of the model and the process of data dimensionality change within the network are detailed in Figure 4. The model is primarily composed of the following three core modules: a CNN feature extractor, a Transformer-based encoder (including multi-head attention and a feed-forward network), and an MLP regression head. Through the synergistic action of these modules, the model can effectively extract light intensity sequence features from the input data and learn the complex relationships between these features, ultimately achieving precise angle prediction.

- CNN Feature Extractor [25,26,27,28]

The CNN feature extraction module is formed by stacking two convolutional layers and max-pooling layers. Each convolutional layer is followed by a batch normalization layer, a ReLU activation function, and a Dropout layer to extract local interference pattern features from the input data and increase the model’s robustness. The max-pooling layer is used to reduce the spatial resolution of the feature maps, decrease the computational load, and extract more representative features. Through multi-layer convolution and pooling operations, the model can effectively capture the dependencies within the interference pattern in the input data. To adapt the feature maps output by the CNN to the Transformer decoder architecture, the model uses a linear layer to transform the dimensionality of the convolutional features into the dimension required by the attention mechanism, thereby better capturing the relationships between features.

- Transformer Encoder [29]

This module is the core of the model, used for modeling the global dependencies within the feature sequence. Since the Transformer’s self-attention mechanism itself lacks the ability to perceive sequence order, this study employs positional encoding using sine and cosine functions. This embeds the absolute or relative position information of each position in the sequence into the feature vectors, enabling the model to perceive positional information. The encoding formulas are as follows:

(25) $$PE\left(pos,2i\right)\;=\;sin(pos\;/\;\left(10000^{2i/d_{model}}\right)),$$

(26) $$PE\left(pos,2i+1\right)\;=\;cos(pos\;/\;\left(10000^{2i/d_{model}}\right)),$$

where PE is the final output positional encoding data, pos represents the position in the sequence, *i* represents the dimension index, and $d_{model}=128$ represents the feature dimension.

Subsequently, by computing multiple attention heads in parallel, the model can capture complex relationships between different positions in the input sequence. Each. attention head independently calculates attention weights and transforms the input sequence into a weighted representation. This paper uses Scaled Dot-Product Attention with the following calculation formula:

(27) $$\mathrm{Attention}\left(Q,K,V\right)\;=\;\mathrm{softmax}\left(\mathrm{QKT}\;/\;\sqrt{dk}\right)V,$$

where Q,K, and *V* are the query, key, and value matrices, respectively, and dk is the dimension of the key vectors.

The final part of the encoder is a feed-forward network. The feed-forward neural network consists of two linear layers and a ReLU activation function, used for non-linear transformation and feature reshaping of the attention mechanism’s output.

- MLP Regression Head

After the Transformer encoding, we obtain a feature sequence containing global information. To perform regression prediction, we first apply global average pooling to all position features in the sequence to get a global feature vector of length 128. Finally, the model uses an Multi-Layer Perceptron (MLP) regression head to map the extracted features to the target angle value. This regression head consists of multiple linear layers, ReLU activation functions, and Dropout layers to achieve precise angle prediction.

#### 2.2.2. Benchmark Models for Comparison

To comprehensively evaluate the proposed CNN–Attention architecture and to understand the contribution of its components, two additional deep learning models were implemented as benchmarks: a standalone Convolutional Neural Network (CNN) and a Long Short-Term Memory (LSTM) network. Both models take the 1000-element intensity sequence as input and output a single angle value.

- CNN Model

This model is designed to assess the effectiveness of convolutional feature extraction alone for AOA estimation. It begins with a feature extractor consisting of two stacked 1D convolutional layers, each followed by ReLU activation, batch normalization, and dropout. A max-pooling layer is applied after the second convolutional block, effectively halving the sequence length. The output features are then transformed by a linear layer to a dimension of 128. To replace the attention mechanism, these features undergo a ReLU activation and are subsequently aggregated across the sequence dimension using global average pooling. Finally, an MLP regression head, comprising three linear layers, ReLU activations, and dropout, predicts the final angle. This architecture closely mirrors the CNN feature extraction and MLP regression head components of our proposed CNN–Attention model, but without the Transformer Encoder module.

- LSTM Model

This model evaluates the capability of recurrent neural networks to capture sequential dependencies in the intensity data. The 1000-element input intensity sequence is first projected to a hidden dimension of 128 via a linear layer. This projected sequence is then fed into a stack of two LSTM layers, each with 128 hidden units. The hidden state from the last time step of the final LSTM layer is extracted and passed to an MLP regression head. This head consists of two linear layers, ReLU activation, and dropout, ultimately outputting the predicted angle.

#### 2.2.3. Peak–Linear Calibration Method

To highlight the advantages of the deep learning method adopted in this paper, we chose a conventional method based on a physical optics model and signal processing techniques as a comparative baseline. The core idea of this method is to utilize the linear correspondence between the position of the principal maximum (i.e., the peak) in the light intensity sequence and the AOA of the spatial light for fitting and prediction [35].

This method mainly includes two stages:

- Peak Position Detection

By setting a specific angle range, it can be ensured that only one principal maximum exists in the light intensity sequence within this range. This allows for the establishment of a one-to-one mapping relationship between the magnitude of AOA and the peak position. By searching and locating the position of the principal maximum peak in the signal, a basis for the model’s subsequent fitting is provided.

- Mapping Relationship and Angle Estimation

Through validation with simulated data, we found that the peak position *x* and the AOA *y* exhibit an approximately linear relationship throughout the measurement range. Therefore, we use a linear function combined with the least squares method to perform a global fit on a set of data to establish the functional relationship between the AOA and the peak position y=f(x). This linear model is represented as y=f(x)=kx+b.

### 2.3. Error Models

In the simulation framework for AOA detection in arrayed waveguides, this study explicitly modeled phase perturbations and manufacturing tolerances to evaluate the sensitivity of the end-to-end output light field intensity sequence to multi-source errors.

#### 2.3.1. Phase Noise

Phase perturbations within the arrayed input waveguide, arising from factors such as temperature drift, mechanical stress, and refractive index fluctuations, are modeled as an additive phase screen. In this simulation, this is primarily implemented by constructing a pixel-wise independent Gaussian white noise phase screen $\phi_{phase}(x$, y)∼N(0, $\sigma_{\phi }^{2})$.

#### 2.3.2. Manufacturing Tolerances

In this work, manufacturing tolerances are broken down into two categories: global or local channel phase drift caused by arrayed input waveguide length errors and the addition of a linear phase gradient within the channel due to arrayed input waveguide pointing deviations.

The length error term is implemented by adding fixed, uniform random noise $\phi_{length}\left(N\right)∼U\left(0,1\right)$ to different channels of the arrayed input waveguide.

The pointing error term is implemented by adding a linear phase gradient to different channels of the arrayed input waveguide, expressed by the formula:

(28) $$\phi_{orientation}\left(x\right)=\;k_{i}\sin\left(\theta_{dev}\right)x,$$

where $k_{i}$ is a proportionality coefficient following a uniform distribution $k_{i}∼U\left(0,1\right)$, and $\theta_{dev}$ is the maximum deviation angle of a single rectangular waveguide in the array. The term $k_{i}\theta_{dev}$ approximates the angle by which the actual rectangular waveguide deviates from its ideal position.

## 3. Results

This section details the setup of the simulation experiments, including the construction of the dataset and parameter selection, and presents the simulation results. We propose an end-to-end regression method based on a CNN–Attention regressor. This method utilizes a convolutional neural network to automatically extract effective features from the data and employs an attention mechanism to focus on important sequence information, thereby achieving more accurate predictions. The performance differences between this method and the peak–linear calibration method will be comprehensively evaluated through comparative experiments.

### 3.1. Simulation Structure Parameters

The simulation parameters are configured as shown in Table 2 below. First, regarding the channel count design, the arrayed input waveguide is set to 5000 to ensure a reasonable optical aperture size. Meanwhile, the number of arrayed output waveguides is set to 1000 to increase the length of the optical intensity sequence, thereby improving the prediction accuracy of the model. In terms of arrayed waveguide dimensions, the spacing between adjacent waveguides is controlled to keep the crosstalk level below 30 dB. The aperture of the arrayed input waveguide is designed to be 1.23 μm to ensure that the waveguide operates in a single-mode transmission state. The aperture of the arrayed output waveguide is designed to be 3 μm to ensure that the receiving waveguide has a high duty cycle.

To simulate the impact of phase noise and manufacturing tolerances, we introduced several error models as described in Section 2.3. For the experiments detailed below, the additive phase noise was modeled with a standard deviation of $\sigma_{\phi }=\frac{\pi }{4}$. The length error term was set to a range corresponding to a phase shift of $[-\frac{\pi }{2}$, $\frac{\pi }{2}]$, and the maximum pointing deviation angle was set to $5^{\circ }$. A detailed analysis of the model’s robustness to phase noise and other parameter variations is provided in Section 3.4. Based on the above design, the coupling efficiency of the arrayed input waveguide for free-space beams with different angles of arrival fluctuates within the range of 61.4% to 81.2%. The arrayed input waveguide coupling efficiency reaches a maximum value of 81.2% when the AOA is $0^{\circ }$. Based on these simulation parameters, the proposed model can achieve high-precision detection of AOAs within a $40^{\circ }$ field of view.

### 3.2. Simulation Results

**Figure 5:**
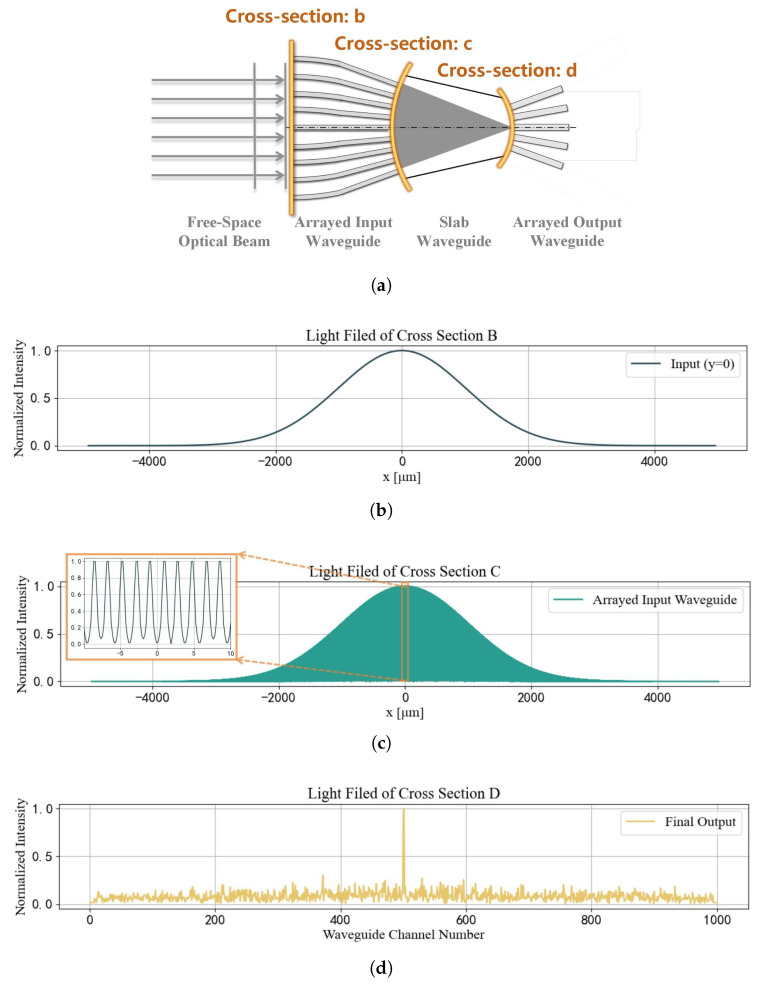
Input and output light field intensity distribution of the arrayed waveguide AOA detection architecture. (**a**) The cross-sectional position of the following light fields in the arrayed waveguides. (**b**) Input light field intensity distribution of the arrayed waveguide structure. (**c**) Output light field intensity distribution of the arrayed input waveguide. (**d**) Output light field intensity distribution of the arrayed output waveguide.

To illustrate the fundamental working principle of the device, we first simulate the light field propagation at a 0° AOA, while including phase perturbation noise and manufacturing tolerances. Figure 5 demonstrates the complete journey of the light field through the core components of the architecture.

The process begins with a Gaussian beam serving as the input light field (Figure 5b), incident on the arrayed input waveguide. After propagating through this array, the continuous light field is discretized into 5000 dense, individual beamlets (Figure 5c). While microscopically discrete, the macroscopic intensity profile of these beamlets retains a Gaussian envelope.

These discrete beamlets then enter the free-propagation region (the slab waveguide implementing the Rowland circle). Finally, upon reaching the focal plane of the Rowland circle, the light converges into a single, focused spot. This spot is then sampled by the arrayed output waveguide, generating a light intensity sequence (Figure 5d). The spatial position of this sequence’s peak is subsequently used to determine the AoA.

**Figure 6:**
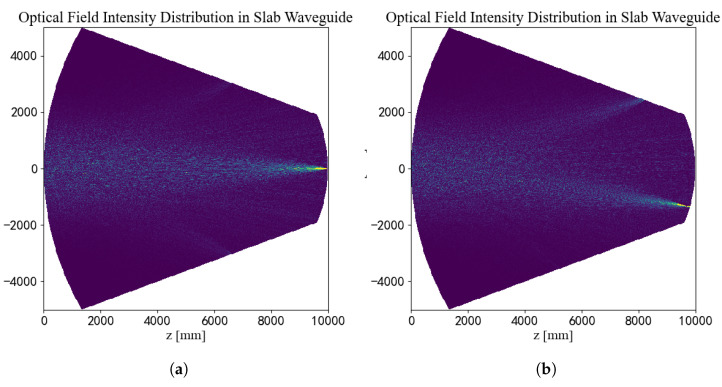
Visualization of the light field intensity evolution within the slab waveguide for different input angles. (**a**) For a 0° AoA, the beam focuses symmetrically onto the center. (**b**) For a 15° AoA, the entire propagation path is tilted, shifting the final focus spot to a corresponding off-center location.

Figure 6 visualizes the light field evolution within the slab waveguide to illustrate the physical mechanism of beam steering, comparing a 0° on-axis with a 15° off-axis incidence. For the 0° AoA (Figure 6a), the light propagates symmetrically to form a focus at the center of the output arc. In contrast, the input wavefront’s phase tilt at 15° AoA (Figure 6b) steers the entire propagation path, resulting in a correspondingly shifted focus spot. This comparison vividly demonstrates the device’s core function: deterministically mapping the input AOA to a unique spatial position.

**Figure 7:**
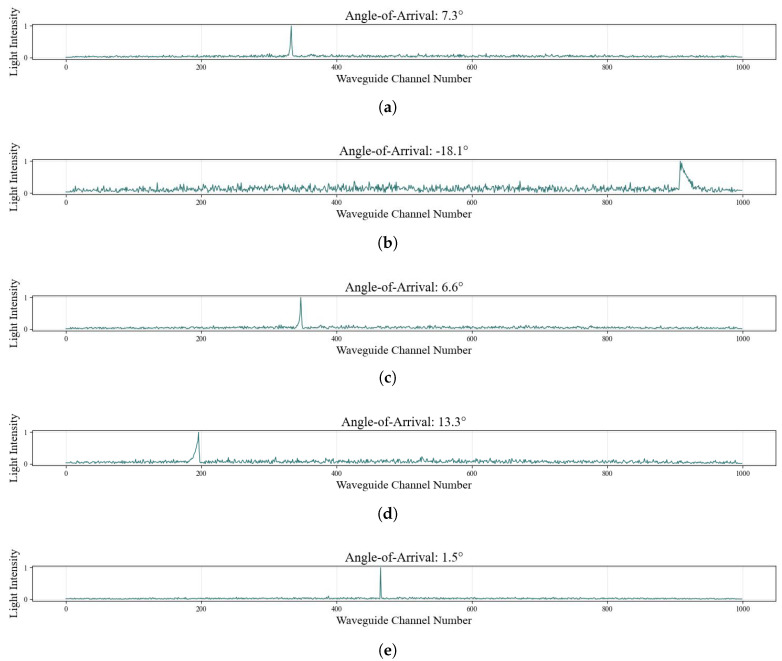
Examples of AOA and arrayed output light field intensity sequences in the arrayed waveguide AOA detection architecture. (**a**) Light intensity sequence at 7.3° AOA. (**b**) −18.1°. (**c**) 6.6°. (**d**) 13.3°. (**e**) 1.5°.

Building on this principle, we selected different AOA values to show the corresponding final output light intensity sequence captured by the arrayed output waveguide, as shown in Figure 7. It can be seen that the position and intensity distribution of the focused spot vary with the angle. The greater the difference in the AOA, the more obvious the difference in the spatial position of the main intensity peak, which is the direct consequence of the beam steering effect illustrated in Figure 6.

### 3.3. AoA Estimation

To evaluate AoA estimation performance, this study compares a traditional signal processing method with a deep learning approach. For this purpose, a total of 2000 datasets were established for training and testing the two methods.

#### 3.3.1. AoA Estimation by Peak–Linear Calibration

The traditional approach investigated is the peak–linear calibration method. This method estimates the AoA by first identifying the waveguide channel with the highest output intensity in the arrayed output waveguide. A linear mapping is then established between the position of this peak intensity channel and the corresponding angle. For this method, which does not require a validation set, the dataset was divided into 1700 samples for the training set and 300 samples for the test set.

**Figure 8:**
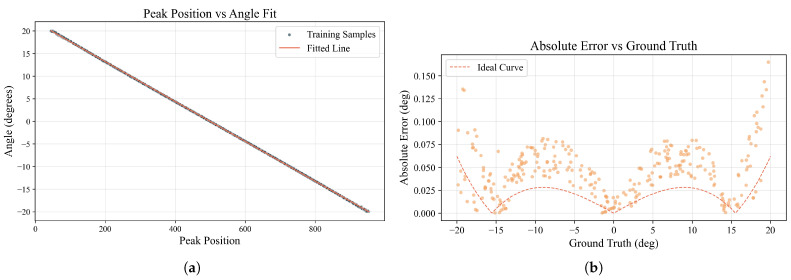
Performance evaluation of the peak–linear calibration method. (**a**) The linear regression fit between the peak channel index and the AoA for the training data. (**b**) The absolute error distribution as a function of the true AoA for the test data, revealing systematic errors at larger angles. The theoretical absolute error curve (dashed line) is also presented, which is derived by applying a linear approximation to the device’s inherently nonlinear grating equation.

The results of this method are summarized in Figure 8. The linear regression performed on the training data, shown in Figure 8a, establishes the mapping function. It exhibits a strong linear correlation, suggesting a good initial fit to the data.

A more detailed analysis of the model’s performance on the test set, illustrated in Figure 8b, reveals inherent operational boundaries. The plot of absolute error versus ground truth AoA exhibits a distinct, symmetrical “W-shaped” dependency. The model achieves its highest accuracy in the low-error troughs of this “W” shape, located at AoAs of approximately 0° and ±15°. In stark contrast, the most significant performance degradation occurs at the extreme edges of the field of view (as AoA approaches ±20°). This nonuniform, angle-dependent accuracy underscores the need for a more sophisticated modeling approach to ensure consistently high performance across the entire operational range.

#### 3.3.2. AOA Estimation by CNN–Attention Regressor

The conventional peak–linear calibration method serves as an effective benchmark, yet to push the boundaries of angle prediction accuracy, we developed a sophisticated deep learning model. This model is a regression-focused Convolutional Neural Network featuring an attention mechanism (CNN–Attention). The dataset for this endeavor was allocated as follows: 1400 samples for training, 300 for validation, and 300 for testing.

**Figure 9:**
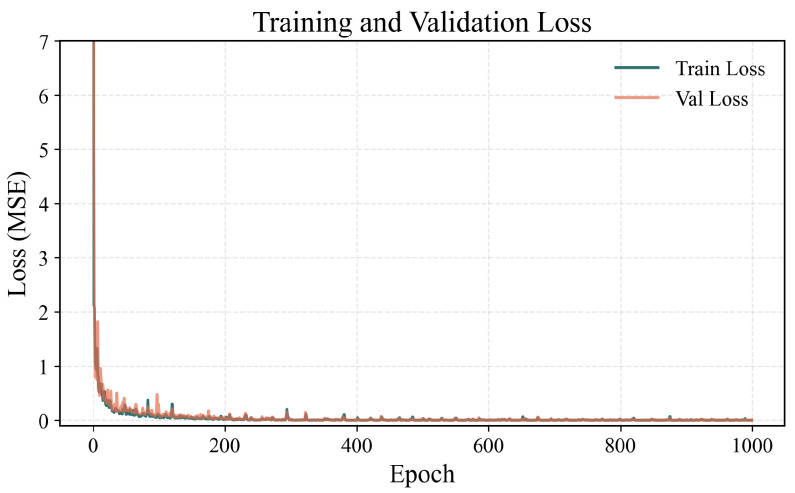
Loss curves for the training and validation sets during model training.

During the model training process, the mean squared error (MSE) was selected as the loss function. To further optimize the training effect of the model, the Adam optimization algorithm was adopted, which can effectively handle non-convex optimization problems. A cosine annealing scheduler was used for the learning rate to smoothly adjust it. By setting the batch size to 32 and the number of training epochs to 1000, the model is ensured to undergo sufficient training, completing the extraction of diffraction pattern features. As shown in Figure 9, the training process was stable, with both training and validation losses steadily converging to a low value. The small gap between the two curves demonstrates the model’s effective generalization and absence of overfitting.

For practical benchmarking, the computational time for training was recorded. The model was trained on a workstation equipped with an Intel Core i9-10900KF CPU (Intel Corporation, Santa Clara, CA, USA), 32 GB of RAM, and an NVIDIA GeForce RTX 3060 (12 GB) GPU (NVIDIA Corporation, Santa Clara, CA, USA). The entire training process for 1000 epochs completed in just 21.73 min. This efficient training time underscores the model’s practicality for rapid development and deployment.

**Figure 10:**
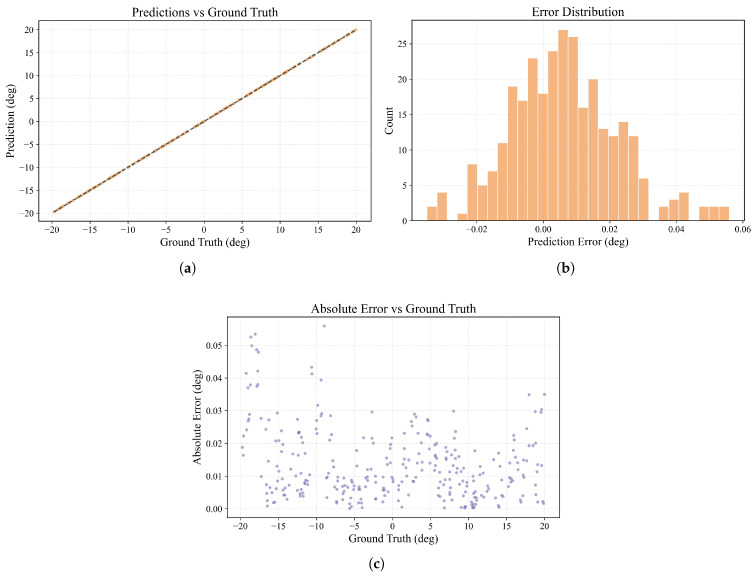
The CNN–Attention regression method angle prediction effect display. (**a**) Predicted vs. Ground truth angles. (**b**) Error distribution histogram. (**c**) Error magnitude distribution across angular range.

The prediction performance of the model is visualized in Figure 10. We obtained high-precision AOA prediction results, with a MAE of 0.0142°, a RMSE of 0.0193°, and a coefficient of determination R^2^ of 0.999997.

#### 3.3.3. Performance Comparison

**Figure 11:**
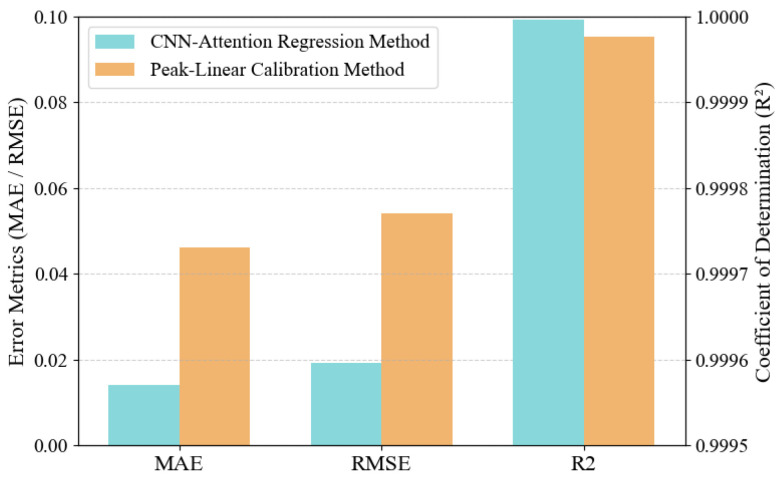
Comparison histogram of key performance indicators of two AOA estimation methods (MAE: Mean Absolute Error; RMSE: Root Mean Square Error; $R^{2}$: Coefficient of determination).

To comparatively analyze the performance of the two methods, we conducted three independent trials. The comparison of the angle prediction performance of the two schemes in different trials is presented in Table 3, and a histogram is used to visually display the angle prediction effect of the two methods, as shown in Figure 11.

From the charts, we can observe that both methods have high R^2^; values, which means that this arrayed waveguide AOA detection architecture has excellent AOA estimation accuracy and robustness, and can accurately recover the initial AOA information of the model from the influence of noise such as phase interference and manufacturing tolerances. Furthermore, we observe that in the three trials, the angle prediction performance of the CNN–Attention regression method is significantly higher than that of the conventional peak–linear calibration method, and it has better effects under different performance evaluation indicators. Finally, in terms of the MAE value, the AOA prediction effect of the deep learning method is 3.25 times that of the conventional method; for the RMSE value, the improvement is 2.81 times.

The selection of the hybrid CNN–Attention architecture is further justified by a detailed comparison against other common deep learning models: a standalone CNN and an LSTM. This analysis, summarized in Table 4, provides a comprehensive evaluation across multiple metrics, including prediction accuracy, model complexity, and computational efficiency.

A clear performance hierarchy emerges from this comparison. The standalone CNN model performed the worst, with a MAE of 0.4122°. While CNNs are excellent at extracting local features from the interference fringes, they struggle to comprehend the global context—that is, the absolute position of the entire fringe pattern, which is the primary indicator of the AOA. This fundamental limitation leads to significantly larger prediction errors.

The LSTM model, which processes the data as a sequence, achieved a much better MAE of 0.0515°. This performance is comparable to that of the direct physics-based peak detection algorithm, suggesting that a standard sequence model can effectively capture the overall shift of the intensity peak.

In stark contrast, the proposed CNN–Attention model proves highly effective by synergizing the strengths of both approaches. The CNN component first extracts rich local pattern features, and the attention mechanism subsequently weighs the importance of these features across the entire sequence. This allows the model to overcome the CNN’s limitation by effectively identifying the global position of the most salient features, resulting in a superior performance that validates our architectural choice.

Notably, our proposed model achieves this state-of-the-art accuracy with a more efficient parameter count than the LSTM. While its inference time of 1.03 ms is marginally higher than the others, it remains well within the threshold for real-time applications, thus offering the best overall balance of accuracy, model efficiency, and practical usability. This result strongly validates our architectural choice.

### 3.4. Robustness Analysis

To validate the model’s suitability for real-world implementation, we performed a comprehensive robustness analysis. This evaluation investigates the model’s resilience against deviations in key operational parameters, structural imperfections arising from fabrication, and noise inherent in signal measurement, directly addressing key practical benchmarks.

#### 3.4.1. Robustness to Phase Noise and Fabrication Imperfections

First, we assessed the model’s resilience to signal noise and common manufacturing tolerances.

- Phase Noise

Our physical simulations indicate that the sampled optical signal maintains a distinguishable structure even when the standard deviation of the phase screen ($\sigma_{\phi }$) is increased to a tested maximum of 0.6π. Under this severe noise condition, the model’s prediction performance degrades, yielding a MAE of 0.704°. While this error is higher than the excellent results achieved at $\sigma_{\phi }=\frac{\pi }{4}$ (reported in Section 3.3.2), it defines the operational limit of the model under extreme phase perturbations.

- Manufacturing Tolerances

We evaluated the model’s performance under the waveguide length errors and pointing deviations defined in our simulation setup. The results confirm that the model maintains high prediction accuracy even in the presence of random length errors (causing phase shifts in the range of $\left[-\frac{\pi }{2},\frac{\pi }{2}\right]$) and pointing deviations up to 5°. This demonstrates that the proposed method effectively compensates for these standard fabrication and alignment imperfections without performance degradation.

#### 3.4.2. Robustness to Operational and Structural Parameter Variations

In addition to the above analysis, we further investigated the model’s generalization capability when faced with parameters outside its training distribution. Specifically, we analyzed its performance under variations in operating wavelength and waveguide spacing ($d_{g}$).

- Wavelength Variation

**Figure 12:**
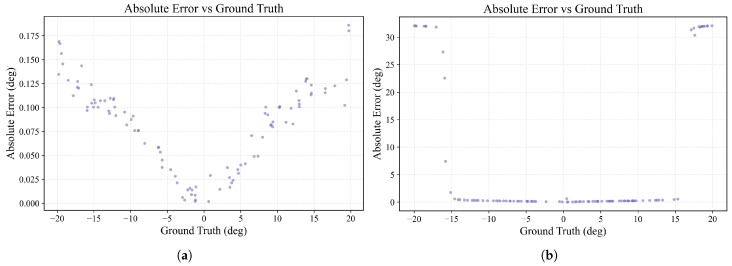
Absolute prediction error under wavelength variations. (**a**) Error distribution with 10% wavelength deviation. (**b**) Error distribution at 1064 nm wavelength, far outside the training distribution. Predictions remain accurate within the ±15°.

A test set was generated using a wavelength that deviated by 10% from the central wavelength used for training. Under this condition, the model achieved a MAE of 0.0775°. As illustrated in Figure 12a, the prediction accuracy is notably higher within a smaller angular range (e.g., ±10°), where the MAE is only 0.0329°. This indicates a strong robustness to moderate wavelength fluctuations.

- Structural Variation

We simulated a 10% deviation in the waveguide spacing to simulate significant structural variations or fabrication errors. The test yielded a remarkably low MAE of 0.0134°. This extremely low error value indicates that our trained model possesses outstanding robustness to changes in waveguide spacing.

It is noteworthy that when parameters like wavelength or waveguide spacing undergo further modification, the effective angular detection range of the device, as defined by Equation (24), also changes. For instance, when we tested the model at a wavelength of 1064 nm, which is far from the training data, we found that angle prediction remained accurate within a ±15° range. However, for angles between 15° and 20°, the model failed to produce valid predictions, as shown in Figure 12b. This confirms that the model’s prediction range is limited by the device’s physical laws, rather than a failure of the model itself.

#### 3.4.3. Summary of Robustness

In summary, these comprehensive tests confirm that our method is not only highly accurate under ideal conditions but also robust against a range of real-world challenges. These include operational parameter shifts (wavelength), structural variations (waveguide spacing), signal noise (phase noise), and other fabrication imperfections (length and pointing errors). This multi-faceted robustness reinforces the model’s strong potential for practical deployment.

## 4. Discussion

This study successfully demonstrates a novel AOA detection method based on arrayed waveguide coherent mode decoding. This method transforms the spatially distributed phase information of a FSO AOA into spatially distributed intensity information and employs an end-to-end regression method based on a CNN–Attention model to recover the beam’s AOA from the intensity sequence. Simulations have verified its excellent performance in high-precision angle prediction, particularly demonstrating strong robustness against various sources of interference.

### 4.1. Underlying Principles of High-Precision Detection

The high-precision AOA prediction performance of this method can be attributed to the synergistic effect of physical optics principles and a deep learning model. From a physical perspective, the core mechanism lies in the multi-beam interference phenomenon guided by the arrayed waveguide structure. When beams from different waveguides are aligned to the same detection position within the slab waveguide, coherent combination occurs, concentrating energy at the center of the principal maximum and forming a distinct principal maximum interference fringe.

On the computational level, the CNN–Attention regression method surpasses traditional methods that rely solely on peak detection. It is capable of learning and extracting complex nonlinear features from the complete signal output by the arrayed output waveguide. This capability allows the model to accurately estimate the AOA even when the signal is distorted by noise. Furthermore, through its powerful feature extraction capabilities, the CNN–Attention regressor can learn to compensate for signal variations caused by specific manufacturing tolerances, thus exhibiting stronger robustness against device fabrication errors.

The systematic “W-shaped” error from the peak–linear method (Figure 8b) is physically explained by the device’s governing grating equation, $\theta_{0}=-\arcsin\left(\sin\theta /n_{s}\right)$ (Equation (19)). This inherent nonlinearity means that forcing a linear fit between the input AOA and the output peak position introduces a predictable, systematic error, whose theoretical shape is shown in Figure 8b. While the linear approximation is adequate near the center of the field-of-view, the pronounced nonlinearity at the angular extremes causes large deviations, resulting in the observed “W-shaped” error pattern. The proposed CNN–Attention regressor, however, is not bound by this linear constraint. It directly learns the complex nonlinear mapping from the intensity sequence, thus avoiding such systematic biases and achieving uniformly high accuracy across the entire detection range.

### 4.2. Advantages and Application Prospects

Compared to traditional AOA detection technologies, the method proposed in this study demonstrates significant advantages and broad application prospects in several aspects. A core contribution is its high potential for miniaturization. Based on mature Planar Lightwave Circuit (PLC) technology [36,37], the entire arrayed waveguide structure can be compactly integrated on a chip, providing a highly integrated AOA estimation solution for portable and embedded applications.

A second key advantage is the significant reduction in system complexity. The system’s optical front-end is entirely passive, obviating the need for the active components (e.g., phase shifters, dynamic beam steering) or complex beamforming networks common in traditional phased arrays. This architecture strategically transfers the primary complexity from the difficult-to-integrate optical domain to the mature and robust domain of digital electronic processing, where an active processing unit executes the decoding algorithms. As a result, this approach not only leads to a substantially simplified optical architecture but also requires only a single, non-recurring calibration, significantly reducing both deployment and operational complexity.

Regarding computational efficiency for real-time systems, our model’s inference time of 1.03 ms is sufficient for many applications like robotic navigation and beam tracking, representing a favorable trade-off between the substantial accuracy gain and modest computational cost. Both its input and output ends are arrayed waveguide structures, giving it great potential for integration with existing waveguide devices like waveguide combiners. This integration requires no additional optical interfaces and holds the promise of using multiple arrayed waveguide AOA detection structures for corresponding channel combinations to enhance optical signal energy for long-range detection. It also offers the possibility of simultaneously realizing multiple functions on a single chip, such as WDM and spatial direction sensing, thus opening new pathways for the construction of next-generation multi-functional photonic integrated systems.

### 4.3. Limitations and Future Work

Despite the promising simulation results, it is important to recognize the applicability boundaries and limitations of the current model, which in turn point to key directions for future research.

#### 4.3.1. Device Specificity and Wavelength Dependency

Our analysis, grounded in the grating equation, reveals that the model’s performance exhibits strong robustness to minor manufacturing variations but requires retraining for systematic changes to core parameters like wavelength. This distinction in sensitivity to geometric parameters versus wavelength has direct implications for device design and generalization. Consequently, future research will prioritize overcoming this wavelength dependency by developing multi-wavelength systems, either through advanced multi-task learning models or innovative broadband waveguide structures.

- Wavelength Dependency and Material Dispersion

The model’s wavelength dependency primarily stems from material dispersion. Although our design utilizes the zeroth-order diffraction (m = 0) where the grating equation $d_{g}\sin\theta +n_{s}d_{g}\sin\theta_{o}=m\lambda$ appears wavelength-independent, the effective refractive index varies with wavelength due to material dispersion. This variation alters the output optical field’s phase profile, consequently affecting prediction accuracy. This explains the observed increase in prediction error when the wavelength deviates from the training value, as detailed in Section 3.4.2.

- Robustness to Waveguide Spacing Variations

In contrast, the model demonstrates exceptional robustness to variations in waveguide spacing $d_{g}$. Under the zeroth-order diffraction condition, the influence of $d_{g}$ is significantly diminished. The neural network has effectively learned a phase-to-angle mapping that is inherently insensitive to $d_{g}$ variations, maintaining high prediction accuracy even with a 10% deviation in $d_{g}$.

- Physical Limits on Model Generalization

However, the model’s generalization capability remains fundamentally constrained by physical principles. The detectable angular range Δθ is ultimately limited by the diffraction limit $\Delta \theta =2\arcsin(\lambda /2d_{g})$. Experimental verification at 1064 nm confirmed accurate predictions within the theoretical range of approximately ±15°, with failure beyond this boundary (Figure 12b). This confirms that the model’s operational limits are physically determined rather than being solely defined by the training data range.

#### 4.3.2. Limitations of the Simplified Simulation Model

The current study relies on a simplified simulation model. A primary limitation, as noted in Section 2, is the use of a 1D simulation model as a first-order approximation. While this approach was instrumental for demonstrating the core principle and training the deep learning regressor, transitioning to a full 2D model and a physical device will introduce several factors that must be considered. Our future work will involve detailed simulations and experimental validation to address these factors. The key aspects are outlined below.

- Asymmetry in Angular Resolution

The current study assumes a 1D variation in AOA. In a practical 2D scenario with both azimuth and elevation angles, the rectangular cross-section of the waveguides (1.23 μm × 1 μm) will lead to different effective mode field diameters in the horizontal and vertical directions. This asymmetry will cause the diffraction-limited spot size at the output to be different for the two axes, likely resulting in non-uniform angular resolution. For instance, the smaller vertical dimension could lead to a larger diffraction angle and thus a lower resolution in the elevation direction compared to the azimuth.

- Increased Crosstalk and Scattering

Transitioning from a 1D linear array to a 2D planar array fundamentally alters the crosstalk environment. In a 2D array, each waveguide is surrounded by a larger number of neighbors compared to the two adjacent ones in a 1D model. While the strongest coupling still occurs between orthogonally adjacent waveguides, the cumulative crosstalk power received by a single waveguide increases due to the contribution from all surrounding neighbors. The overall increase in integrated crosstalk can lead to a higher optical noise floor, potentially broadening the main interference peak at the output and reducing the signal-to-noise ratio. Furthermore, out-of-plane scattering losses, an inherent 3D effect primarily caused by sidewall roughness [38], are not captured by the 1D model and could reduce the overall device efficiency in a physical implementation.

#### 4.3.3. Unmodeled Physical Effects and Environment Factors

While our proposed model demonstrates robustness against fabrication tolerances, a comprehensive analysis requires the consideration of additional physical phenomena and environmental factors. In this section, we address these potential deviations and discuss strategies to mitigate them in practical experiments.

- Power Fluctuations and Propagation Loss

To evaluate the model’s robustness against material absorption and scattering losses, we performed a supplementary simulation incorporating a typical propagation loss of 1 dB/cm. Additionally, to account for optical power instability, we introduced multiplicative noise drawn from a Gaussian distribution (μ=1, σ=0.05) to the power of each output channel. The resulting MAE for AOA estimation was 0.0168°, which is only marginally higher than the ideal case of 0.0142°. This result demonstrates the resilience of the proposed method, indicating that the model effectively learns the relative intensity distribution rather than relying on absolute power levels.

- Incoherent Background Light

In practical environments, incoherent background light may degrade the signal-to-noise ratio. This influence can be suppressed using standard signal processing techniques. For instance, by modulating the input optical signal, lock-in amplification can be employed to effectively distinguish the coherent signal from static background noise [39]. This strategy will be integral to the design of subsequent experiments.

- Nonlinear Effects

Nonlinear optical effects are currently not included in the model, as the sensing application is designed to operate at low optical power levels where such phenomena are typically insignificant. However, ensuring the device operates within its linear regime is crucial; therefore, verifying the linear dynamic range will be a priority in future experimental characterization.

- Polarization State

The polarization state of the light beam can impact performance due to fabrication-induced birefringence, a well-known challenge in integrated photonics. To ensure polarization-independent operation in future implementations, we plan to adopt established solutions [21], such as designing waveguides with a square cross-section to minimize birefringence, applying dispersion compensation, or utilizing polarization diversity schemes.

#### 4.3.4. Practical Implementation and Fabrication Challenges

Finally, the physical implementation of the device on a silicon-based or silicon nitride platform presents further practical challenges. The following discussion outlines the proposed implementation route and an analysis of the expected challenges, which will form the basis of our future experimental research.

- Fabrication Process and Tolerance Effects

We propose fabricating the device on a silicon nitride (${\mathrm{Si}}_{3}N_{4}$-on-${\mathrm{SiO}}_{2}$) platform, which is well-suited for this application due to its low propagation loss and broad transparency window. The fabrication can be accomplished using standard silicon photonics manufacturing techniques [40,41,42,43,44]: (1) Starting with a silicon wafer with a thick thermal oxide (${\mathrm{SiO}}_{2}$) bottom cladding. (2) Depositing a ${\mathrm{Si}}_{3}N_{4}$ core layer via LPCVD or PECVD. (3) Defining the waveguide pattern using EBL for prototyping or DUV lithography for mass production. (4) Transferring the pattern to the ${\mathrm{Si}}_{3}N_{4}$ layer using RIE. (5) Encapsulating the device with a ${\mathrm{SiO}}_{2}$ upper cladding.

However, real-world fabrication is subject to tolerances that will affect device performance. The manufacturing tolerances modeled in our simulation (phase perturbations, length and pointing errors) are direct consequences of physical process variations. For instance, non-uniformity in lithography and etching can lead to variations in waveguide width and height. These dimensional deviations alter the effective refractive index, which in turn affects the phase accumulation and modifies the interference condition. This can cause a shift in the focal spot position and a degradation of the interference fringe contrast. Similarly, sidewall roughness from etching is a primary source of propagation loss and random phase errors [38]. A key objective of our future experimental work will be to characterize the impact of these fabrication-induced non-uniformities and validate the robustness of our CNN–Attention regressor, which is designed to learn and compensate for such systematic deviations.

- Expected Optical Loss

The total on-chip optical loss is another critical parameter to be determined experimentally. We anticipate several major sources of loss [40,41,44,45]: (1) Propagation loss, standard LPCVD processes for stoichiometric ${\mathrm{Si}}_{3}N_{4}$ consistently yield propagation losses in the range of 0.1 to 1.0 dB/cm at the 1550 nm wavelength. Moreover, values below 0.1 dB/cm are routinely achievable through process optimization such as high-temperature annealing. (2) Coupling loss, our simulations in Section 3.1 quantify this input coupling efficiency, which is inherently dependent on the AOA. The efficiency ranges from a maximum of 81.2% (a loss of 0.9 dB) at the center of the field of view to 61.4% (a loss of 2.1 dB) at the edges. In addition to this primary input loss, there are also smaller internal transition losses at the interfaces where light couples from the input waveguides into the slab waveguide, and subsequently from the slab into the output waveguides. (3) Bending loss, which is expected to be negligible given the large radius of curvature in our design. The characterization and minimization of these losses will be a central part of the device’s experimental validation.

## 5. Conclusions

This research aims to address the growing demand for miniaturized, high-precision AOA detection in modern optical detection systems. We innovatively propose and theoretically validate a novel AOA detection scheme based on coherent mode decoding with arrayed waveguides. The study confirms a precise, one-to-one correspondence between the AOA of the incident light beam and the main peak position of the decoded output light intensity, which establishes a solid physical foundation for high-precision angle retrieval using data-driven methods. To achieve high-precision AOA detection, this study designs and employs an end-to-end regression model that combines a convolutional neural network with an attention mechanism. Simulation results strongly demonstrate the effectiveness of this method, achieving MAE and RMSE as low as 0.0142° and 0.0193°, respectively. This level of precision is significantly superior to conventional methods based on peak detection and linear calibration, highlighting the powerful capability of deep learning models in accurately resolving complex optical patterns. Furthermore, the method exhibits excellent robustness under simulated phase noise and fabrication tolerance-induced perturbations, demonstrating its great potential for practical applications in the future.

The contribution of this research lies in providing a viable path for on-chip integrated AOA detection systems that combine high precision, low complexity, and high robustness, thereby advancing the interdisciplinary field of photonics and artificial intelligence. Looking ahead, our top priority is to finalize the device fabrication and experimental validation. Building upon this foundation, we will focus on optimizing both the decoding algorithm and the system architecture for multi-beam AoA detection. These efforts are aimed at unlocking its full application potential in fields such as LiDAR, LWS and optical communications.

## Figures and Tables

**Table 1:** Main structural parameters and their meanings for the arrayed waveguide AOA detection model.

Symbol	Parameter Explanation
λ	Wavelength of the optical signal
θ	AOA of the incident beam
$w_{g}$	Core width of the arrayed input waveguide
$h_{g}$	Core thickness of the arrayed input waveguide
$d_{g}$	Center-to-center spacing of adjacent arrayed input waveguides
$N_{g}$	Number of arrayed input waveguides
$L_{0}$	Length of the central channel arrayed input waveguide
*R*	Focusing length of the slab waveguide (Rowland circle diameter)
$w_{o}$	Core width of the arrayed output waveguide
$h_{o}$	Core thickness of the arrayed output waveguide
$d_{o}$	Center-to-center spacing of adjacent arrayed output waveguides
$N_{o}$	Number of arrayed output waveguides

**Table 2:** Simulation structure parameter values for arrayed waveguide AOA estimation model.

Symbol	Parameter Name	Parameter Value
λ	Optical Signal Wavelength	1550 nm
θ	Incident Beam AOA	($-20^{\circ }$, $20^{\circ }$)
$w_{g}$	Arrayed Input Waveguide Core Width	1.23 µm
$h_{g}$	Arrayed Input Waveguide Core Thickness	1 µm
$d_{g}$	Arrayed Input Waveguide Center Spacing	1.91 µm
$N_{g}$	Number of Arrayed Input Waveguides	5000
$L_{0}$	Center Channel Arrayed Input Waveguide Length	500 µm
*R*	Rowland Circle Diameter	10 mm
$w_{o}$	Arrayed Output Waveguide Core Width	3 µm
$h_{o}$	Arrayed Output Waveguide Core Thickness	1 µm
$d_{o}$	Arrayed Output Waveguide Center Spacing	3.68 µm
$N_{o}$	Number of Arrayed Output Waveguides	1000
*z*	Gaussian Beam Propagation Distance	1 m
$b_{w}$	Gaussian Beam Waist Radius	2 mm
-	Upper Cladding Material	${\mathrm{SiO}}_{2}$
-	Core Material	${\mathrm{Si}}_{3}N_{4}$
-	Substrate Material	${\mathrm{SiO}}_{2}$

**Table 3:** Comparison of evaluation metrics across different methods. MAE, RMSE, and R^2^ stand for Mean Absolute Error, Root Mean Square Error, and the coefficient of determination, respectively.

Evaluation Metrics	MAE		RMSE		R^2^	
Method	Peak–Linear Calibration Method	CNN–Attention regression method	Peak–Linear Calibration Method	CNN–Attention regression method	Peak–Linear Calibration Method	CNN–Attention regression method
Trial 1	0.0461	0.0134	0.0542	0.0173	0.999977	0.999998
Trial 2	0.0461	0.0153	0.0542	0.0214	0.999977	0.999996
Trial 3	0.0461	0.0140	0.0542	0.0193	0.999977	0.999997
Mean	0.0461	0.0142	0.0542	0.0193	0.999977	0.999997

**Table 4:** Detailed comparison of different deep learning architectures.

Model	MAE (°)	RMSE (°)	Parameters (k)	Training Time (s/Epoch)	Inference Time (ms/Sample)
CNN	0.4122	0.6040	80.96	0.24	0.98
LSTM	0.0515	0.0696	272.77	1.18	0.69
CNN–Attention (Proposed)	0.0142	0.0193	213.44	1.30	1.03

## Data Availability

The simulation data supporting the findings of this study are available within the article.
